# Sex-Specific Improvements in Myocardial Function and Angiogenesis with SGLT-2 Inhibitor Canagliflozin in a Swine Model of Metabolic Syndrome

**DOI:** 10.3390/ijms26051887

**Published:** 2025-02-22

**Authors:** Dwight D. Harris, Mark Broadwin, Christopher Stone, Sharif A. Sabe, Meghamsh Kanuparthy, Ju-Woo Nho, Kelsey C. Muir, M. Ruhul Abid, Frank W. Sellke

**Affiliations:** Division of Cardiothoracic Surgery, Department of Surgery, Cardiovascular Research Center, Alpert Medical School of Brown University, Brown University Health, 2 Dudley Street, MOC 360, Providence, RI 02905, USA; ddharris@bidmc.harvard.edu (D.D.H.); mark.broadwin@gmail.com (M.B.); christopher_stone@brown.edu (C.S.); ssabe@bidmc.harvard.edu (S.A.S.); meghamsh_kanuparthy@brown.edu (M.K.); ju-woo_nho@brown.edu (J.-W.N.); kelsey_muir@brown.edu (K.C.M.); ruhul_abid@brown.edu (M.R.A.)

**Keywords:** ameroid constrictor, canagliflozin, chronic myocardial ischemia, female, male, metabolic syndrome, sex, sodium-glucose cotransporter-2 inhibitor

## Abstract

There is a significant body of literature to suggest that coronary artery disease (CAD) is a highly sex-specific disease. The study of sex-specific therapeutics and sex-specific responses to treatment for CAD remains underreported in the literature. Sodium-glucose transporter 2 (SGLT2) inhibitors are of growing interest in the treatment of ischemic heart disease and heart failure; however, the sex-specific response to SGLT2 inhibitors is unknown. We studied an SGLT2 inhibitor, canagliflozin, in a swine model of metabolic syndrome (MS) and chronic myocardial ischemia with emphasis on the sex-specific outcomes. Yorkshire swine (*n* = 21) were obtained at 6 weeks of age and fed a high-fat diet to induce MS. Left thoracotomy was performed on all swine at 11 weeks of age for the placement of an ameroid constrictor to model chronic myocardial ischemia. Swine recovered for two weeks, then were assigned to either the drug group, CAN 300 mg daily group (M = 5, F = 5), or the control group (CON, M = 5, F = 6). Both groups received 5 weeks of therapy. After completion of therapy, swine underwent functional assessment and terminal harvest. The male animals treated with CAN (CAN-M) had significant increases in stroke volume and cardiac output (*p* = 0.047, *p* < 0.001) compared to control males (CON-M), which were not seen in females treated with CAN (CAN-F) compared to control females (CON-F). Effective arterial elastance was decreased in CAN-M compared to CON-M. The CAN-F group had a significant increase in ischemic myocardial capillary density compared to CON-F (*p* = 0.04). There was no difference in capillary density between the CAN-M and CON-M groups. CAN treatment resulted in sex-specific changes in angiogenesis and myocardial function. The CAN-M group had significant improvements in cardiac function based on afterload reduction, stroke volume, and increased cardiac output not seen in the CAN-F group. The CAN-F group had increased ischemic myocardial capillary density. These findings provide a foundation for further investigation of the sex-specific effects of SGLT-2 inhibitors in humans.

## 1. Introduction

Ischemic heart disease remains a leading cause of mortality worldwide and results in significant morbidity and health care expenditure [1]. There have been many advances in the treatment of heart failure and ischemic heart disease; however, therapeutic options are still limited for patients with advanced heart disease who are not candidates for surgical or percutaneous intervention [2]. This group of patients is often relegated to maximal medical therapy. The definition of maximal medical therapy is rapidly changing, and one class of medications that has gained particular attention is sodium-glucose cotransporter-2 (SGLT-2) inhibitors [3,4].

SGLT-2 inhibitors are primarily used in the treatment of diabetes mellitus and primarily function by inhibiting the coupled reabsorption of glucose from the proximal tubule of the nephron [5]. In addition to their glucose-lowering properties, SGLT-2 inhibitors decrease cardiovascular mortality, heart failure mortality, atrial fibrillation, and heart failure readmissions [6,7,8,9,10,11,12,13]. Given the growing body of clinical data favoring the use of SGLT-2 inhibitors in cardiovascular disease, they have gained a 1A recommendation from the American Heart Association for use in heart failure [3].

Though there is growing clinical data to support the use of SGLT-2 inhibitors in cardiovascular disease, the biochemical mechanism is not fully understood [7,14]. Basic science studies utilizing mice and zebrafish models of acute ischemia have demonstrated improved cardiac function with SGLT-2 inhibitor treatment [15,16,17,18]. Our group has previously studied the SGLT-2 inhibitor canagliflozin (CAN) using a swine model of chronic myocardial ischemia with and without metabolic syndrome [19,20,21]. We found that in a normal diet model, SGLT-2 inhibition increased cardiac function, including cardiac output, myocardial perfusion, and improved diastolic function [19].

This prior work has greatly expanded our insight into the effects of SGLT-2 inhibitors in the myocardium, but it fails to account for how a modern high-fat diet and metabolic syndrome (MS) influence SGLT-2 inhibitor function in the myocardium. Repeating the normal diet experiments with a high-fat diet known to induce vascular dysfunction similar to MS showed a significant decrease in pulse pressure and increase in cardiac output and capillary density [22]. This study greatly expanded our understanding of how CAN functions in the myocardium in the setting of a real-world diet; however, it did not account for the sex-specific response to SGLT-2 inhibitors.

It is well known that cardiovascular disease results in sex-specific phenotypes, with females experiencing less disease burden; however, female patients often present at later ages and have worse outcomes [23,24,25]. Females are known to have a higher symptom burden and a two times higher risk of mortality. This is likely related to several metabolic and physiologic factors. Estrogen is believed to exert protective effects against atherosclerosis in premenopausal women; however, estrogen hormone therapy can be harmful, particularly after a period of estrogen deprivation, indicating the protective effect is related to more than estrogen levels [23,25]. This is further validated by the finding that transgender women receiving estrogen hormone therapy have increased the risk of myocardial infarction compared to cisgender women but not cisgender men [23,26]. Females also have smaller epicardial coronary arteries, increased endothelial shear stress, higher baseline blood flow, and more microvascular defects [23,27,28]. Females are believed to have increased microvascular dysfunction [23,24]. Furthermore, there is a large discrepancy in the sex-specific study of cardiovascular therapeutics [23]. It has previously been shown in humans that males benefit more from SGLT-2 inhibition, and large animal studies have shown similar findings, with males benefiting more than females [29,30]. We have previously validated this finding by showing that male swine treated with SGLT-2 inhibition have increased cardiac function compared to female swine treated with SGLT-2 inhibition [30].

The objective of this study is to investigate the sex-specific response to SGLT-2 inhibition in the setting of chronic myocardial ischemia (CMI) and a high-fat diet using our previously validated swine model and proteomic analysis.

## 2. Results

### 2.1. Functional Results

**Figure 1:**
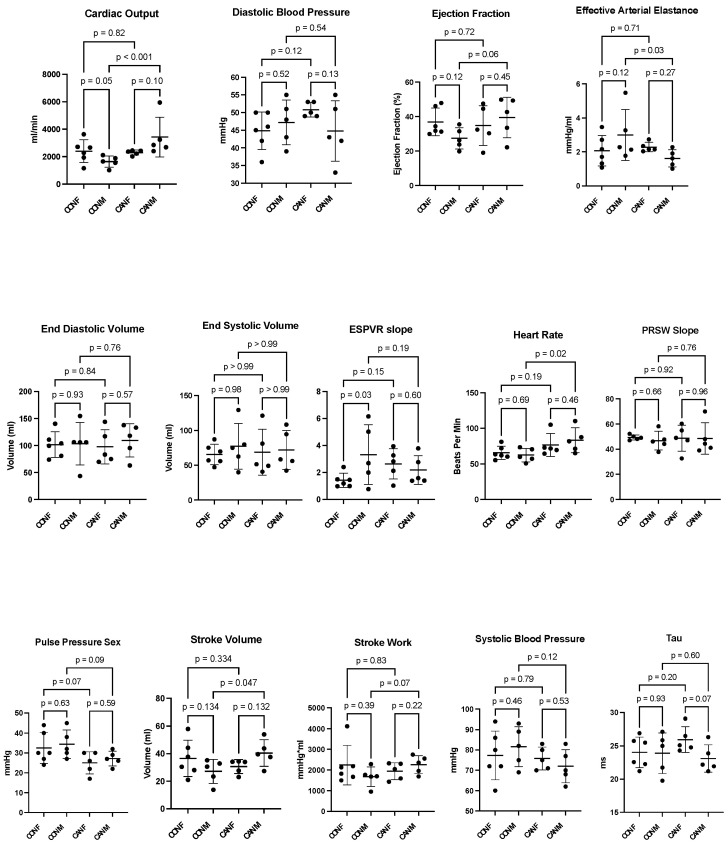
Functional Data. There was a significant increase in stroke volume, heart rate, and cardiac output in canagliflozin males (CAN M) compared to control males (CON M), which was not seen in canagliflozin females (CAN F) compared to control females (CON F). There was a significant decrease in effective arterial elastance in CAN M compared to CON M. There was a trend towards increased ejection fraction and stroke work in the CAN M group. There was a trend towards decreased pulse pressure in both the CAN M and CAN F groups. There were no significant changes in Tau, end-systolic pressure-volume relationship (ESPVR) slope, preload recruitable stroke work (PRSW) slope, end-diastolic volume, end-systolic volume, diastolic blood pressure, or systolic blood pressure.

There was a statistically significant increase in stroke volume, heart rate, and cardiac output in the CAN-M group compared to the CON-M group, which was not seen in CAN-F compared to the CON-F group (all *p* < 0.05, Figure 1, Table 1). There was a statistically significant decrease in effective arterial elastance in CAN-M compared to CON-M, which was not seen in CAN-F compared to CON-F (*p* = 0.03, Figure 1, Table 1). There was a trend towards increased ejection fraction and stroke work in the CAN-M group (*p* = 0.06, *p* = 0.07, Figure 1). There was a trend towards decreased pulse pressure in both the CAN-M and CAN-F groups (*p* = 0.09, *p* = 0.07, Figure 1, Table 1). There were no significant changes in Tau, end-systolic pressure-volume relationship (ESPVR) slope, preload recruitable stroke work (PRSW) slope, end-diastolic volume, end-systolic volume, diastolic blood pressure, or systolic blood pressure (all *p* > 0.05, Figure 1, Table 1).

### 2.2. Myocardial Angiogenesis and Perfusion

**Figure 2:**
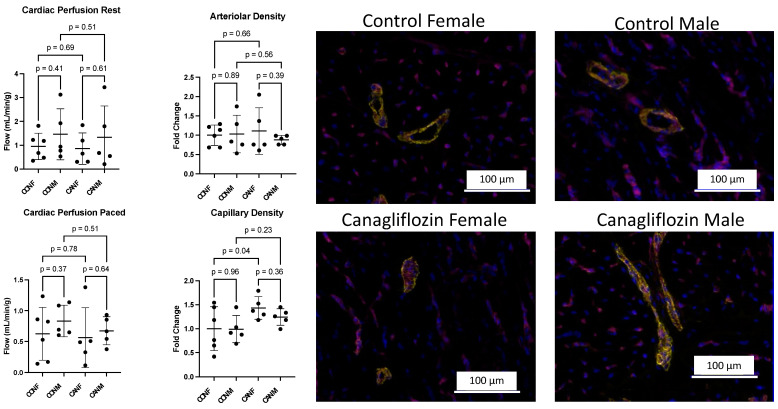
Myocardial Angiogenesis and Perfusion. There was a statistically significant increase in capillary density by isolectin b4 (magenta) in canagliflozin females (CAN F) compared to control females (CON F), which was not seen in canagliflozin males (CAN M) compared to control males (CON M). There was no significant difference in resting myocardial perfusion, stressed myocardial perfusion with pacing at 150 beats per minute, or in arteriolar density by alpha smooth muscle actin (yellow).

There was a statistically significant increase in capillary density in CAN-F compared to CON-F, which was not seen in CAN-M compared to CON-M (*p* = 0.04, Figure 2, Table 1). There was no significant difference between groups in myocardial perfusion at rest or while stressing the heart by pacing at 150 beats per minute. Arteriolar density was not significantly different between groups (all *p* > 0.05, Figure 2, Table 1).

### 2.3. Proteomic Analysis

**Figure 3:**
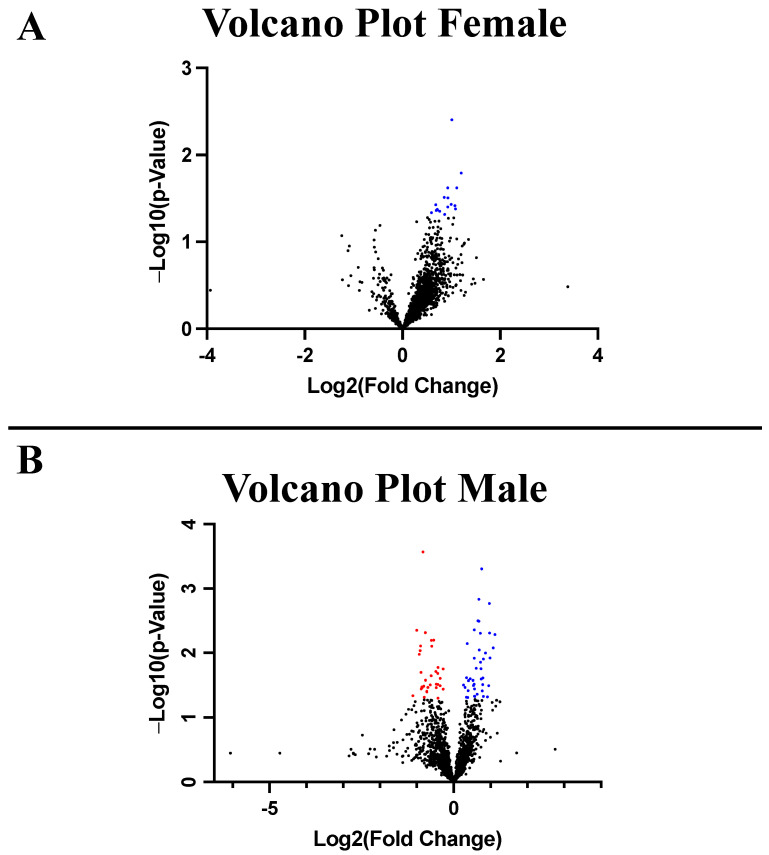
Volcano Plots. (**A**) is the volcano plot for canagliflozin females compared to control. There were no significantly decreased proteins and 26 significantly increased proteins in the canagliflozin females compared to control. (**B**) is the volcano plot for canagliflozin males compared to control. There were 42 significantly decreased and 60 significantly increased proteins in the canagliflozin males group compared to control. Blue represents proteins with a significant increase, and red represents proteins with a significant decrease.

**Figure 4:**
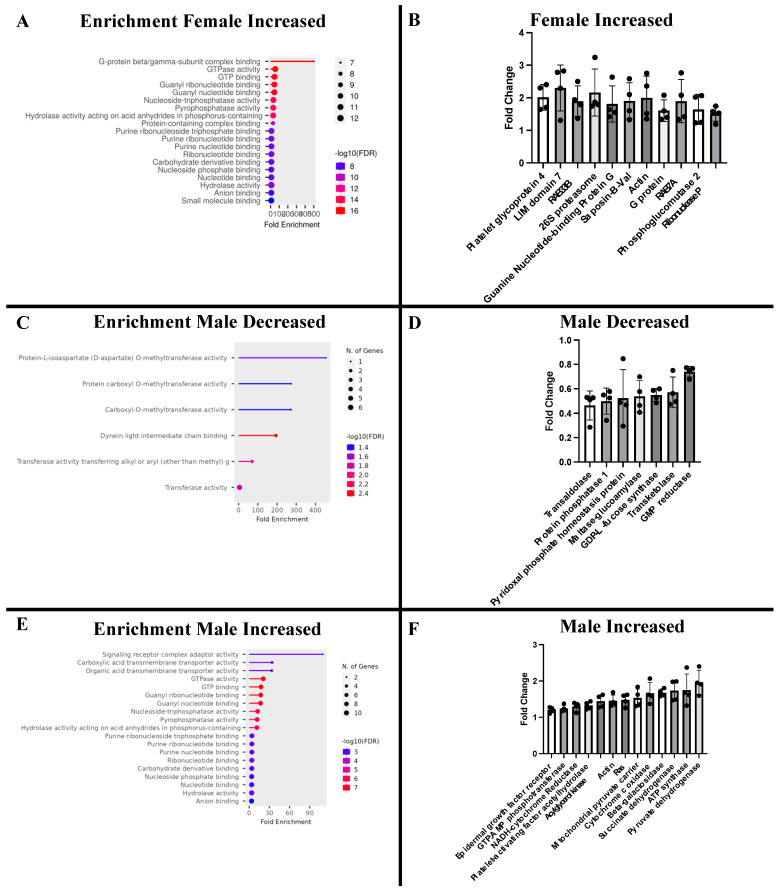
Enrichment Analysis and Select Proteins. (**A**). Female-increased proteins enrichment analysis. (**B**). Female-increased select proteins. (**C**). Male-decreased proteins enrichment analysis. (**D**). Male-decreased select proteins. (**E**). Male-increased proteins enrichment analysis. (**F**). Male-increased select proteins.

Proteomic analysis identified approximately 2900 total proteins. There were no significantly decreased proteins in the CAN-F group compared to CON-F (Figure 3A). There was a significant increase in 26 proteins in the CAN-F group compared to CON-F (Figure 3A). There were 42 significantly decreased proteins in the CAN-M group compared to CON-M (Figure 3B). There was a significant increase in 60 proteins in the CAN-M group compared to CON-M (Figure 3B). Enrichment analysis of significantly increased proteins in the CAN-F group showed a significant increase in protein metabolism pathways (Figure 4). Enrichment analysis of significantly decreased proteins in CAN-M showed decreases in metabolic transferases (Figure 4). Enrichment analysis of significantly increased proteins in the CAN-M group showed a significant increase in protein and carbohydrate metabolism pathways (Figure 4), including an increase in electron transport chain proteins (Figure 4).

## 3. Discussion

The findings of this study greatly expand our understanding of the sex-specific responses to SGLT-2 inhibition in the setting of CMI in a high-fat diet model of MS. 

Although there is growing clinical data to support the use of SGLT-2 inhibitors in cardiovascular disease in general, the biochemical mechanism is not fully understood, and the sex-specific response to SGLT-2 inhibitors remains underrepresented in the literature [29]. The limited data extant prior to this experiment suggest that female patients benefit less than male patients treated with SGLT-2 inhibitors [29]. Our previous work with swine using a normal-diet model validated many of the findings seen in human clinical studies showing that male swine treated with SGLT-2 inhibitors exhibited improved cardiac function compared to their female counterparts [30]. However, metabolic syndrome (MS) is becoming an increasingly common comorbidity that significantly influences the effectiveness of therapeutics for ischemic heart disease [31,32]. 

The CAN-M group had significant improvements in cardiac output and stroke volume, which were not seen in the CAN-F group. This was combined with a significant decrease in effective arterial elastance, a marker for afterload reduction. There were also notable trends towards increased stroke work and ejection fraction in the CAN-M group. These results are similar to our prior study using a normal diet, which showed that CAN-M had improved functional parameters not seen in CAN-F, and with human studies that showed males benefited more than females treated with SGLT-2 inhibitors [29,30]. This is likely related to an increase in many key metabolism and oxidative phosphorylation pathways. This is evident in our proteomic analysis, as we found significant increases in proteins such as pyruvate dehydrogenase, cytochrome c oxidase, and ATP synthase in the male treatment group. Pyruvate dehydrogenase has been shown to be an important marker for healthy metabolism, and increasing ATP synthase has been identified as a potential target in the treatment of myocardial ischemia [33,34].

We hypothesize that the significant increases in proteins such as pyruvate dehydrogenase, cytochrome c oxidase, and ATP synthase observed in the male treatment group enhance myocardial fuel supply and metabolism, leading to an increase in stroke volume and, consequently, a higher ejection fraction. This rise in stroke volume, in turn, contributes to an overall increase in cardiac output. Conversely, we propose that the absence of metabolic changes in the female group accounts for the lack of observed physiological effects. Further studies are needed to explore how sex-specific hormones and genetic factors influence this process.

There was a significant increase in capillary density in CAN-F compared to CON-F. There was no increase in perfusion in the CAN-F or CAN-M groups, making it unclear if the change in capillary density is meaningful. However, it is possible that the study timeline or methods limit the detection of changes in perfusion. This finding is different from our normal-diet study in two keyways. First, our work with normal-diet swine failed to show a change in capillary density [30]. Secondly, there was no change in myocardial perfusion in males or females treated with CAN compared to the increase in perfusion in CAN-M in our prior study [30]. These findings imply that the high-fat diet is modulating the effect of SGLT-2 inhibitors in the myocardium and possibly decreasing the effectiveness of SGLT-2 inhibitors. However, it is possible that the 5-week post-treatment time point of the study is too early to detect changes in perfusion related to changes in vascular density. Further studies are needed with multiple time points to see if the improvements in vascular density contribute to an augmentation of myocardial perfusion. The addition of vascular reactive studies could also help further characterize the microenvironment and potential benefits of angiogenesis.

Ultimately, the results of our study suggest that both CAN-M and CAN-F may benefit from SGLT-2 inhibitors; however, there appear to be sex-specific benefits to CAN therapy. CAN-M appears to have more significant improvements in cardiac function, while CAN-F shows improvements in myocardial vascularity. This study further validates the clinical use of SGLT-2 inhibitors and provides a basis for further investigation in human studies.

This study greatly increased our understanding of the complex interactions between SGLT-2 inhibitor CAN, MS, and sex in the context of a clinically relevant model of chronic myocardial ischemia. However, it is important to consider several key limitations. The study contains a relatively small sample size of 5–6 in each group of animals. Previously published literature suggest that this sample size is more than sufficient for the use of sophisticated proteomic methods, but the study may be underpowered to detect changes in more subtle physiological parameters. Moreover, the study is limited to one time point and one fixed dose, which could result in the failure to detect changes that would occur at longer time points or with higher concentrations. Finally, this study only analyzes the effects of one SGLT-2 inhibitor, CAN, and may not be applicable to all SGLT-2 inhibitors.

## 4. Methods

### 4.1. Swine Model

**Figure 5:**
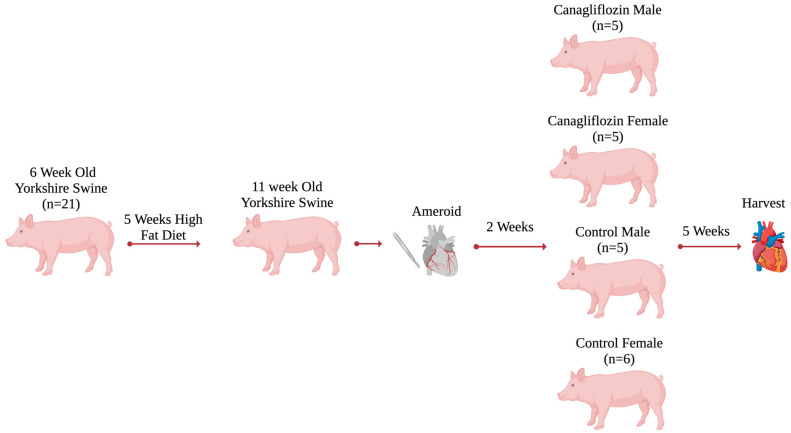
*Methods.* Yorkshire swine (*n* = 21) were obtained at the age of six weeks and fed a high-fat diet to model MS. Chronic myocardial ischemia was induced at the age of 11 weeks by placing an ameroid constrictor. After two weeks of recovery, swine were assigned to either the control group (CON, F = 6, M = 5) or CAN 300 mg daily (F = 5, M = 5). After 5 weeks of therapy, all swine underwent terminal harvest procedures, including functional measurements, a myocardial perfusion assessment, and myocardial tissue collection.

Yorkshire swine (*n* = 21) (Cummings School of Veterinary Medicine of Tufts University Farm, Grafton, MA, USA) were obtained at the age of six weeks and fed a 500 g/d high-cholesterol diet comprised of 2.3% corn oil, 4% cholesterol, 1.5% sodium cholate, 17.2% coconut oil, and 75% regular chow (Sinclair Research, Columbia, MO, USA) to model MS [22]. Chronic myocardial ischemia was induced at the age of 11 weeks by placing an ameroid constrictor (Research Instruments SW, Lebanon, OR, USA) around the left coronary circumflex artery (LCx) [22]. After two weeks of recovery, swine were assigned to either the control group (CON, F = 6, M = 5) or CAN 300 mg daily (F = 5, M = 5 Janssen Pharmaceuticals, Beerse, Belgium) [22]. All swine underwent terminal harvest procedures, including functional measurements, a myocardial perfusion assessment, and myocardial tissue collection after 5 weeks of treatment (Figure 5).

### 4.2. Sample Size and Dosing

Using the mean differences and standard deviations for cardiac output and ejection fraction from our prior study on normal diets studying sex-specific response to CAN, a minimum sample size was calculated for physiologic studies using the equation n=2(2.8)^2(standard devation)2(mean difference )2  [35]. This suggested a minimum of 2 animals per group. The required sample size for proteomics and similar methods is a matter of debate, but many would agree that 5 per group would be an acceptable minimum for this type of study. Given the cost of the experiment, sample size calculations, and the need of 5 per group for proteomics, the decision was made to include a minimum of 5 animals per group in the study [36,37].

CAN 300 mg daily is the maximum dose used in host human patients. As a result, 300 mg daily was used in our initial CAN studies, and we have previously shown that at a dose of 300 mg daily, CAN results in substantial improvements in swine cardiac function [19,22,30].

### 4.3. Animal Care

All swine received humane care in compliance with current ethical standards, including the Guide for the Care and Use of Laboratory Animals [22]. All experiments were reviewed and approved by the Institutional Animal Care and Use Committee of Rhode Island Hospital (Protocol #505821, 23 November 2021) [22].

### 4.4. Ameroid Constrictor Procedure

The swine received oral aspirin (10 mg/kg) and oral cephalexin (30 mg/kg) one day preoperatively and five days postoperatively as previously reported [22,38]. Anesthesia was induced with intramuscular telazol (4.4 mg/kg) and xylazine (2.2 mg/kg) as previously reported [22,38]. Endotracheal intubation was performed, and anesthesia was maintained with inhaled isoflurane [22,38]. The swine was placed in a modified right lateral decubitus position [22,38]. The surgical site was prepped in the typical sterile fashion with betadine. The chest was accessed with a mini-thoracotomy on the left side in the 3rd intercostal space [22,38]. The pericardium was opened, and the left atrium was retracted to expose the left anterior descending artery (LAD) and LCx [22,38]. The LCx was exposed using sharp and blunt dissection [22,38]. The LCx dissection was performed to take off the LCx from the left main coronary artery to create consistent areas of ischemia [22,38]. The LCx was isolated with a vessel loop. Systemically, heparin (80 IU/kg) was given to prevent coronary thrombosis [22,38]. The vessel loop was used to occlude the LCx for 2 minutes while simultaneously injecting 5 mL of gold-labeled microspheres (BioPal, Worcester, MA, USA) into the left atrial appendage. The ameroid constrictor was placed around the LCx. Nitroglycerin (2cc) was sprayed on the artery to prevent vasospasm. The pericardium was closed with absorbable sutures as previously reported [22,38]. The chest and skin were closed in layers as previously reported [22,38]. Post-op pain was controlled with a fentanyl patch (4 μg/kg), and intramuscular buprenorphine (0.03 mg/kg) was administered before the closure of the chest as previously reported [22,38].

### 4.5. Harvest Procedure

Anesthesia and pre-op care are the same as previously described above in Section 4.3. The swine was placed supine, and the surgical area was prepped with betadine. The thoracic cavity was accessed by median sternotomy. The pericardium was opened and adhesion lysed to expose the right atrium, left atrium, and apex [22,38]. The left femoral artery was exposed by direct cutdown. The swine was given a bolus (80 IU/kg) of intravenous heparin. The groin was cannulated with a 7fr catheter using the Seldinger technique [22,38]. Myocardial perfusion was assessed by injecting 5 mL of isotope-labeled microspheres (BioPal, Worcester, MA, USA) into the left atrium and simultaneously withdrawing 10 mL of blood from the femoral catheter [22,38]. This was performed at rest and while stressing the heart by pacing at 150 beats per minute [22,38]. Ventricular function was assessed using a pressure-volume catheter (Transonic, Ithica, NY, USA) introduced directly into the left ventricular apex as previously reported [22,38]. After completion of physiological testing, anesthesia was increased, and the heart was excised as previously reported [22,38]. The heart was dissected to remove epicardium and myocardial fat. The tissue was divided into segments based on location with respect to the LCx and LAD and flash frozen in liquid nitrogen as previously reported [22,38].

### 4.6. Immunofluorescence

α-smooth muscle actin (SMA) and isolectin B4 staining was performed on tissue from the most ischemic myocardial area and imaged by iHisto (iHisto, Salem, MA, USA) [22]. Slides were reviewed and analyzed using QuPath (University of Edinburgh, Edinburgh, Scotland, UK). Capillary density was calculated by determining the percentage of tissue area positive for isolectin B4 [22]. QuPath’s automated detection algorithm was applied to three randomly selected 1 mm^2^ sections per slide as previously described [19]. Arteriolar count was conducted using QuPath’s automated detection algorithm. Three 1 mm^2^ sections were randomly selected, and the number of SMA-positive objects with a minimum size of 100 μm^2^ per area of tissue section were counted as previously described [19]. The triplicate was averaged across slides for both stains.

### 4.7. Data Analysis and Statistics

All data was analyzed utilizing Prism 10 (GraphPad Software, San Diego, CA, USA). Data was tested for normality with a Shapiro–Wilk test. Nonparametric data was analyzed with a Mann-Whitney U test, and parametric data was analyzed with a Student’s *t*-test. Capillary density and arteriolar count are represented as mean fold change normalized to the average female control. Outliers larger than two standard deviations from the mean were excluded. Probability values less than 0.05 were considered significant.

### 4.8. Proteomics

The proteomics for this study was conducted by the proteomics core facility at the University of Massachusetts Boston. Proteomic analysis was performed on myocardial samples from the nonischemic LAD territory and the most ischemic area. Please see supplement one for a detailed account of the proteomics used in this study (Supplementary Materials).

Proteomic analysis was only preformed on common proteins found in all samples. Probability values less than 0.05 were considered significant regardless of fold change. Pathway analysis was conducted with ShinyGO 0.76 (South Dakota State University, Brookings, SD, USA).

## 5. Conclusions

This manuscript summarizes the first attempt to study the complex interplay of sex, metabolic disease, ischemic heart disease, and SGLT-2 inhibition. Male swine with ischemic heart disease and metabolic dysfunction treated with CAN exhibited improved cardiac function, as evidenced by afterload reduction, increased stroke volume, and higher cardiac output. This was accompanied by an upregulation of several metabolic pathways in the CAN-M group that were not observed in CAN-F. In contrast, female swine with ischemic heart disease and metabolic dysfunction treated with CAN did not exhibit the same physiological benefits seen in males; however, there was an increase in capillary density in the ischemic myocardium of the CAN-F group. These findings suggest that CAN treatment induces sex-specific changes in angiogenesis and myocardial function. The results of this study provide potential targets for further investigation in both animal models and human subjects treated with SGLT-2 inhibitors. Additionally, these findings may help guide future research into sex-specific therapeutics for ischemic heart disease.

## Figures and Tables

**Table 1:** Functional Perfusion and Vascular Data. Table 1 summarizes the means and standard deviation (SD) for the data in Figure 1 and Figure 2. End-systolic pressure-volume relationship (ESPVR) slope, preload recruitable stroke work (PRSW) slope.

Functional Data	Control Females	Control Males	Canagliflozin Females	Canagliflozin Males
Cardiac Output (mL/min, mean ± SD)	2397 ± 830	1643 ± 412	2295 ± 177	3426 ± 1440
Diastolic Blood Pressure (mmHg, mean ± SD)	44.8 ± 5.3	47.2 ± 6.3	50.8 ± 2.0	44.8 ± 8.6
Ejection Fraction (%, mean ± SD)	36.9 ± 8.0	27.4 ± 6.1	34.7 ± 11.5	39.5 ± 11.7
Effective Arterial Elastance (mmHg/mL, mean ± SD)	2.1 ± 0.9	3.1 ± 1.5	2.3 ± 0.3	1.6 ± 0.5
End-Diastolic Volume (mL, mean ± SD)	101.7 ± 23.6	103.3 ± 39.5	97.9 ± 31.8	109.6 ± 30.7
End-Systolic Volume (mL, mean ± SD)	65.4 ± 14.7	77.3 ± 32.9	68.7 ± 33.0	71.9 ± 28.0
ESPVR Slope (slope, mean ± SD)	1.4 ± 0.52	3.3 ± 2.2	2.6 ± 1.1	2.2 ± 1.1
Heart Rate (bpm, mean ± SD)	65.7 ± 9.3	62.3 ± 9.4	76.7 ± 16.1	83.2 ± 17.4
PRSW Slope (slope, mean ± SD)	49.3 ± 2.0	46.7 ± 7.4	48.7 ± 10.4	48.5 ± 12.5
Pulse Pressure (mmHg, mean ± SD)	32.5 ± 7.8	34.4 ± 7.2	25.0 ± 5.6	27.2 ± 3.7
Stroke Volume (mL, mean ± SD)	36.5 ± 13.2	27.2 ± 8.7	30.7 ± 5.1	40.5 ± 4.3
Stroke Work (mmHg*mL, mean ± SD)	2237 ± 958	1675 ± 475	1939 ± 408	2259 ± 443
Sytolic Blood Pressue (mmHg, mean ± SD)	77.3 ± 12.0	81.6 ± 9.8	75.8 ± 5.6	72.0 ± 8.2
Tau (ms, mean ± SD)	24.0 ± 2.3	23.9 ± 3.1	26.0 ± 1.9	23.1 ± 2.1
Perfusion Data				
Cardiac Perfusion Rest (mL/min/g, Mean ± SD)	0.95 ± 0.55	1.46 ± 1.07	0.86 ± 0.66	1.33 ± 1.32
Cardiac Perfusion Paced (mL/min/g, Mean ± SD)	0.63 ± 0.43	0.83 ± 0.26	0.56 ± 0.48	0.67 ± 0.23
Vascular Density				
Arteriolar Density (fold change, mean ± SD)	1.0 ± 0.2	1.0 ± 0.4	1.1 ± 0.5	0.9 ± 0.1
Capillary Density (fold change, mean ± SD)	1.0 ± 0.4	1.0 ± 0.3	1.4 ± 0.2	1.2 ± 0.2

## Data Availability

Data are available upon request to the corresponding author.

## Supplementary Materials

The supporting information can be downloaded at: https://www.mdpi.com/article/10.3390/ijms26051887/s1.

## Abbreviations

ESPVR	End-systolic pressure-volume relationship
CAN	Canagliflozin
CAN-F	Canagliflozin females
CAN-M	Canagliflozin males
CMI	Chronic myocardial ischemia
CON-F	Control females
CON-M	Control males
PRSW	Preload recruitable stroke work
