# Supplementary material for: Sex-Specific Improvements in Myocardial Function and Angiogenesis with SGLT-2 Inhibitor Canagliflozin in a Swine Model of Metabolic Syndrome
Source: Int J Mol Sci. 2025 Feb 22;26(5):1887. doi: 10.3390/ijms26051887 (PMC11900068; doi:10.3390/ijms26051887)
Supplement: Supplementary file 1 [file ijms-26-01887-s001.zip › ijms-3451519-supplementary.pdf]

Supplement 1 proteomics methods:

Tissue samples were weighed and crushed into a powder using liquid nitrogen. The powdered tissue was resuspended in T-PER (Thermo Fisher Scientific, Waltham, MA, USA) with Halt Protease Inhibitor tablet (Thermo Fisher Scientific, Waltham, MA, USA) and PhosSTOP (Sigma-Aldrich, St. Louis, MO, USA). A bicinchoninic acid (Thermo Fisher Scientific, Waltham, MA, USA) assay was performed to determine protein concentration. Aliquots of 100 µg were buffer exchanged via addition of 4x by volume chilled acetone. The samples were precipitated overnight at - 20 °C. The proteins were pelleted via centrifugation at 16,000 x g for 10-minutes. The protein pellets were rinsed with 50 µL of 90% chilled acetone and recollected via centrifugation at 16,000 x g for 5-minutes. The supernatants were discarded, and the protein pellets were air dried for 5-minutes. The samples were resuspended in 100 mM ammonium bicarbonate, reduced with 2.1 µL of 500 mM Dithiothreitol [incubated for 45-minutes at 60 °C], alkylated with 11.5 µL of 500 mM Indole-3-acetic acid [incubated at RT in the dark for 30-minutes], and digested overnight with 2.5 µL of 1 mg/mL Trypsin/Lys-C at 37 °C. One microliter of each sample was extracted and combined.

A UltiMate 3000 HPLC (Thermo Fisher, San Jose, CA, USA) was used for 2D fractionation. The HPLC was operated at a flow rate of 0.060 mL/min using the following gradient: starting at 5% B, 0-60 minutes 0-50% B, 60-65 minutes 50-100% B, 65-75 minutes 100% B, 75-80 minutes 100-5% B, 80-90 minutes 5% B, for column re-equilibration. Mobile phase solvents were purchased from Fisher Chemical (Fair Lawn, NJ, USA) and were Optima LC/MS Grade. Mobile phase A was 96:4 water/acetonitrile with 5 mM ammonium formate. Mobile phase B was 80:20 acetonitrile/water with 5 mM ammonium formate. The sample was separated on a Waters Acquity UPLC BEH C18 column (1.7 µm particle size, 1.0 mm x 100 mm, Waters, Milford, MA, USA). A total of 40 fractions were concatenated into 20 for a duration of 60 seconds each between minutes 20-60 of the gradient.

The samples were then analyzed via LC-IM-MS/MS with an Evosep One (Evosep, Odense, Denmark) coupled online to a Bruker timsTOF HT mass spectrometer (Bruker Scientific LLC, Billerica, MA). The fractionated samples were processed via data-dependent acquisition in order to generate a data-

sourced library. The unfractioned samples were then analyzed via data-independent acquisition and searched against the proper spectral library. Of each sample, an approximately 500 ng was loaded onto an EvoTip for injection. Raw data files were processed with PaSER and Bruker Proteoscape (BPS) with a false discovery rate of 1%.

Peversed-phased nano-liquid chromatography Electrospray ionization- mass spectrometry (RP-nLC/ESI-MS) conditions data-dependent acquisition:

The fractions were run on an Evosep One nLC coupled online to a Bruker timsTOF HT mass spectrometer. The samples were run on the Evosep 30 samples per day (SPD) gradient (Evosep, Odense, Denmark) on a PepSep Endurance column (15 cm x 150 mm, 1.9 µm) (PepSep, Odense, Denmark). The timsTOF was operated in PASEF mode with a scan range of 100-1700 m/z and a mobility range of 0.60-1.60 V·s/cm3. The ramp time and accumulation times were both set to 100.0 ms. The duty cycle was set to 100.0%, ramp rate to 9.42 Hz, and the MS averaging to 1. For the MS/MS parameters, the number of PASEF ramps was set to 10 with a total cycle time of 1.17 s. The charge minimum was set to 0 and the maximum to 5. The target intensity was set to 10000 and the intensity threshold was set to 2500. The collision energies were set to 20.00 eV for 1/K0 0.60 and 59.00 eV for 1.60 1/K0.

RP-nLC/ESI-MS conditions data-independent acquisition:

The samples were run on an Evosep One (Evosep, Odense, Denmark) nLC coupled online to a Bruker timsTOF HT mass spectrometer (Bruker Scientific LLC, Billerica, MA). The samples were run on the Evosep 30 samples per day (SPD) gradient (Evosep, Odense, Denmark) on a PepSep Endurance column (15 cm x 150 mm, 1.9 µm) (PepSep, Odense, Denmark). The timsTOF was operated in dia-PASEF mode with a scan range of 100-1700 m/z and a mobility range of 0.60-1.60 V·s/cm3. The ramp time and accumulation times were both set to 100.0 ms. The duty cycle was set to 100.0%, ramp rate to 9.42 Hz, and the MS averaging to 1. For the MS/MS parameters, the mass range was set to 400-1201 Da and the mobility range was set to 0.60-1.43 1/K0 with an estimated cycle time of 1.80 s. The collision energies

were set to 20.00 eV 1/K0 0.60 and 59.00 eV and for 1.60 1/K0. A total of 16 PASEF events were measured across 32 windows that covered a mobility range of 0.3 1/K0 and mass range of 26 m/z. The first PASEF event covered mobilities 0.6-0.9 and 0.9-1.2 and masses 400-426 and 800-826 m/z. The second PASEF event covered mobiles mobilities 0.62-.092 and 0.92-1.22 and masses 425-451 and 825-851 m/z. This pattern repeated for all 32 windows/16 PASEF scans.

Data-dependent Acquisition Data Analysis/Spectral Library Generation:

Protein identification on the fractions of the combined sample were done with BPS (Bruker Scientific LLC, Billerica, MA, USA) using ProLuCID 1,2, DTASelect2 3 4 and Census 5,6. Mass spectra were streamed via the PaSER plugin directly from the timsTOF's acquisition control software (timsControl) to the PaSER workstation via a dedicated LAN connection and were searched against Sus scrofa protein database (downloaded on 22 August 2023) plus sequences of known contaminants such as keratin and porcine trypsin concatenated to a decoy database in which the sequence for each entry in the original database was reversed 11 using ProLuCID. 20 ppm precursor tolerance and 30 ppm fragment ion tolerance were used. Search space included all fully-/half-tryptic peptide candidates with 2 missed cleavages. Carbamidomethylation (+57.02146) of cysteine was set as a static modification and we require 1 peptide per protein and at least one tryptic terminus for each peptide identification. Additionally, phosphorylation (+79.9663) of serine, threonine, and tyrosine, oxidation (+15.994) of methionine was considered as a variable modification and up to 2 variable modifications were allowed per peptide. TIMScore was appended to raw search results to allow to use the peptide Collisional Cross Section (CCS) during the validation process. These search results were validated, assembled, and filtered using the DTASelect program (version 2.1) with false discovery rate (FDR) of 0.01; under such filtering conditions, the estimated false discovery rate was below ~1% at the protein level in all analysis with peptide FDR < 0.01. These data were used to generate a spectral library with PaSER. The settings on the library generation were max delta ppm of 15.0.

Data-independent Acquisition Data Analysis with BPS

Protein identification and quantification analysis were done with BPS (Bruker Scientific LLC, Billerica, MA, http://www.bruker.com) using TIMS DIA-NN. Mass spectra were streamed via the PaSER plugin directly from the timsTOF's acquisition control software (timsControl) to the BPS workstation via a dedicated LAN connection and pre-processed into a binary file for consumption by TIMS DIA-NN. A spectral library consisting of 69,797 precursors, including peptide modifications such as oxidized methionine and phosphorylated serine, threonine, and tyrosine was re-annotated against SwissProt Sus scrofa protein database (downloaded on 22 August 2023). 20 ppm precursor tolerance and 15 ppm fragment ion tolerance were used along with Top 3 precursors for quantitation. Multiple samples were assembled and match-between-runs performed to fill-in missing values with an outlier frequency of 0.2 following which global normalization was performed.

Data-dependent Acquisition Data Analysis with PEAKS

Protein identification and quantification were done by PEAKS Studio (11.5.6 Build, Bioinformatics Solutions Inc., Waterloo, Ontario, Canada). PEAKS data refine and database search nodes were used. The data were refined using default settings (mass correction and associating features with chimera check boxes were checked). For the database search, a precursor mass tolerance of 20.0 ppm and fragment ion tolerance of 0.05 Da were employed. The enzyme was set to Trypsin (semi specific) with a maximum number of missed cleavages per peptide set to 2. Static Modifications - Carbamidomethylation (+57.02146) on C, and N-termini and Variable Modifications – Acetylation (+42.0106) on N-termini and Oxidation (+15.9949) on M were applied. A maximum of 2 PTMs per peptide were allowed. The data were searched against Sus scrofa protein database (downloaded on 15 Mar. 2024) and cRAP contaminants database. The Deep Learning Boost checkbox was checked. Peptide lengths were restricted between 6 and 45. All 64 DDA samples were processed together and the data were exported as a spectral library.

Data-independent Acquisition Analysis with PEAKS

Protein identification and quantification were done by PEAKS Studio (11.5.6 Build, Bioinformatics Solutions Inc., Waterloo, Ontario, Canada). PEAKS spectral library, database search, and LFQ nodes were used. The spectral library node was used with a precursor tolerance of 20 ppm, fragment ion tolerance of 0.5 Da, a CCS error tolerance of 0.05, and with the optimize tolerance checkbox checked. The generated spectral library was used. The database node with the enzyme was set to Trypsin and the following modifications were added: Static Modification - Carbamidomethylation (+57.02146) on C and Variable Modifications – Acetylation (+42.0106) on N-termini, and Oxidation (+15.9949) on M. A maximum of 1 PTM per peptide was allowed. The data were searched against Sus scrofa protein database (downloaded on 15 Mar. 2024) and the cRAP Contaminant Database. Peptide lengths were restricted between 7 and 30, fragment m/z were restricted between 200 and 1800, precursor m/z were restricted between 300 and 1800, and charge states were restricted between 1 and 4. The quantification node was used to generate specified comparisons. Samples were grouped according to condition and/or gender. Match Between Runs was set to auto-detect with a feature intensity greater than or equal to 100. The ion mobility tolerance was set to 0.05. Data were normalized to the total ion count.
